# Antibiotic treatment using amoxicillin-clavulanic acid impairs gut mycobiota development through modification of the bacterial ecosystem

**DOI:** 10.1186/s40168-023-01516-y

**Published:** 2023-04-10

**Authors:** Madeleine Spatz, Gregory Da Costa, Rebecka Ventin-Holmberg, Julien Planchais, Chloé Michaudel, Yazhou Wang, Camille Danne, Alexia Lapiere, Marie-Laure Michel, Kaija-Leena Kolho, Philippe Langella, Harry Sokol, Mathias L. Richard

**Affiliations:** 1 Micalis Institute, INRAE, Université Paris-Saclay, 78352 Jouy-en-Josas, AgroParisTech France; 2 Paris Center for Microbiome Medicine, Fédération Hospitalo-Universitaire, Paris, 75012 France; 3 Faculty of Medicine, Human Microbiome Research Program, University of Helsinki, 00014 Helsinki, Finland; 4 Folkhälsan Research Center, 00250 Helsinki, Finland; 5 Children’s Hospital, Helsinki University, 00029 Helsinki, Finland; 6 Department of Pediatrics, Tampere University, 33520 Tampere, Finland; 7 Gastroenterology Department, Centre de Recherche Saint-Antoine (CRSA), Saint Antoine Hospital, INSERM, Sorbonne Université, AP-HP, Paris, 75012 France

**Keywords:** Microbiota, Mycobiota, Antibiotics, Enterobacteriaceae

## Abstract

**Background:**

Effects of antibiotics on gut bacteria have been widely studied, but very little is known about the consequences of such treatments on the fungal microbiota (mycobiota). It is commonly believed that fungal load increases in the gastrointestinal tract following antibiotic treatment, but better characterization is clearly needed of how antibiotics directly or indirectly affect the mycobiota and thus the entire microbiota.

**Design:**

We used samples from humans (infant cohort) and mice (conventional and human microbiota-associated mice) to study the consequences of antibiotic treatment (amoxicillin-clavulanic acid) on the intestinal microbiota. Bacterial and fungal communities were subjected to qPCR or 16S and ITS2 amplicon-based sequencing for microbiota analysis. In vitro assays further characterized bacterial-fungal interactions, with mixed cultures between specific bacteria and fungi.

**Results:**

Amoxicillin-clavulanic acid treatment triggered a decrease in the total fungal population in mouse feces, while other antibiotics had opposite effects on the fungal load. This decrease is accompanied by a total remodelling of the fungal population with the enrichment in *Aspergillus*, *Cladosporium*, and *Valsa* genera. In the presence of amoxicillin-clavulanic acid, microbiota analysis showed a remodeling of bacterial microbiota with an increase in specific bacteria belonging to the Enterobacteriaceae. Using in vitro assays, we isolated different Enterobacteriaceae species and explored their effect on different fungal strains. We showed that *Enterobacter hormaechei* was able to reduce the fungal population in vitro and in vivo through yet unknown mechanisms.

**Conclusions:**

Bacteria and fungi have strong interactions within the microbiota; hence, the perturbation initiated by an antibiotic treatment targeting the bacterial community can have complex consequences and can induce opposite alterations of the mycobiota. Interestingly, amoxicillin-clavulanic acid treatment has a deleterious effect on the fungal community, which may have been partially due to the overgrowth of specific bacterial strains with inhibiting or competing effects on fungi. This study provides new insights into the interactions between fungi and bacteria of the intestinal microbiota and might offer new strategies to modulate gut microbiota equilibrium.

Video Abstract

**Supplementary Information:**

The online version contains supplementary material available at 10.1186/s40168-023-01516-y.

## Background

In the last 15 years, the intestinal microbiota has indeed been identified as a cofactor in diseases of the digestive tract (inflammatory bowel diseases (IBDs) or colorectal cancer) but also in diseases indirectly related to the gut, such as allergies, diabetes, and obesity [1, 2]. Gut microbiota members other than bacteria, such as fungi, viruses, and archaea, have been neglected for a long time. Nonetheless, these minority microorganisms have also been associated with various pathologies, particularly intestinal inflammation. Indeed, it has been shown that there is fungal dysbiosis in Crohn’s disease patients, and that gut fungal strains can be associated with IBDs or cancer development [3–7].

Nevertheless, the balance between bacterial and fungal commensal populations remains poorly described; most data report interactions between two single fungal and bacterial species; and mostly pathogen microorganisms, but very rarely, to our knowledge, describe the crosstalk level of an entire ecosystem or characterize the abundance of fungi [8]. The effects of antibiotics on the bacterial microbiota are particularly well described, but very little is known about the impact on the fungal microbiota (mycobiota). The first study to investigate fungal modifications during antibiotic treatment described an increase in the *Candida* genus, while the Enterobacteriaceae family was decreased, but this work was performed using culture-dependent techniques [9]. In a mouse model of antibiotic treatment, mice with oral gavage of *Candida albicans* had an antibiotic-dependent response for the colonization of this yeast, with an increase in the *C. albicans* population (especially after treatment with ceftriaxone, cefoperazone, or ticarcillin-clavulanic acid) in most cases [10]. The same scientists found similar results in a clinical study also focusing on *C. albicans* colonization [11]. Among the studies now focusing on the mycobiota, the majority found that fungal growth was favored in the mouse gut after treatment with different antibiotics, such as cefoperazone or a cocktail of ampicillin, neomycin, vancomycin, and metronidazole [12, 13]. In a recent clinical study following several antibiotic treatments during *Clostridioides difficile* infections (metronidazole, fidaxomicin, and vancomycin), Lamendella and coworkers [14] described specific modifications of bacterial and fungal populations dependent on the drugs used but gave no information on the modification of the global fungal abundance.

Here, we studied the effect of a broad-spectrum antibiotic commonly used in humans (amoxicillin-clavulanic acid) and provided a comprehensive analysis of fungal and bacterial microbiota modifications. We showed that amoxicillin-clavulanic acid strongly affected fungi of the gut microbiota in a mouse model of antibiotic treatment and particularly decreased their total abundance, while it was commonly accepted that antibacterial treatments had the opposite effect [10, 11, 13]. This decrease was also concomitant with the increase in specific bacteria, such as the Enterobacteriaceae family. Analysis of a cohort of infant gut samples treated with amoxicillin without clavulanic acid confirmed that this antibiotic treatment decreased fungal load. Specific in vitro and in vivo analyses in mice allowed the identification of potential bacterial strains that may impair fungal growth. This study has therefore deepened our knowledge of the interactions between bacteria and fungi within the host and has contributed to our understanding of this complex and dynamic ecosystem.

## Materials and methods

### Patient characteristics

We collected fecal samples from antibiotic-naïve infants starting at admission due to an infection with respiratory syncytial virus (RSV) as previously reported [15, 16]. Here, we used 19 samples from 7 infants, the same 7 infants before (considered controls) and after receiving amoxicillin because of otitis media as a complication of RSV. Fecal samples were collected before and during amoxicillin treatment and at different timepoints (from 1 to 5 days) (Table 1). The samples were immediately frozen at −20 °C and transported frozen at −70 °C until analyzed. The median age of the infants included was 2—4 months (median, range 0.8–7.0, all males).

**Table 1:** Number of infants stratified by control and amoxicillin, divided into time

**Timepoints**	**Before (day 0)**	**During (days 1–2)**	**During (days 3–5)**
Control (no. of infants—7; no. of samples—7)	7	0	0
Amoxicillin (no.of infants—7; no. of samples—12)	0	7	5

### Mice

A 6-week-old female C57BL/6J mice were purchased from Janvier Laboratory (Le Genest, France) and used 1 week after delivery. Animals were kept in humidity- and temperature-controlled rooms under a 12-h light-dark cycle and had access to a chow diet and water ad libitum. All experiments were performed in accordance with the ethics committee “Comite d’Ethique en Experimentation Animale” (COMETHEA C2EA — 45, Jouy en Josas, France). Every experiment was repeated at least two times.

### Fecal microbiota transfer (FMT)

Mice received feces from a healthy donor as previously described [17]. Briefly, feces from humans were recovered and immediately stored at 4 °C in an anaerobiosis generator (Genbox, Biomérieux, Capronne, France) to favor the preservation of anaerobic bacteria. Samples were processed within 24 h in a Coy chamber. The feces were rapidly diluted 100-fold in brain-heart infusion (BHI, Becton Dickinson) supplemented with 0.5 mg/ml L-cysteine (Sigma‒Aldrich, St. Louis, MO, USA) and 20% skim milk (Becton Dickinson) (vol/vol) and stored in aliquots at −80 °C. This ready-to-use fecal suspension was used for FMT to mice.

Mice were fasted for 1 h and then subjected to bowel cleansing by oral-gastric gavage with PEG (polyethylene glycol, Macrogol 4000, Fortrans, Ipsen Pharma, France). Four hours later, mice received human feces by oral gastric gavage (350 μl of resuspended feces prepared as described above). Mice were then allowed free access to food and water. FMT was repeated once a week for 3 weeks, before the antibiotic or before and during the bacterial administration. Bowel cleansing was only performed on day 21.

### Gavage with fungi and bacteria

*Candida albicans* SC5314 (ATCC, Molsheim, France) was used in this study. The yeast was grown on yeast extract peptone dextrose (YEPD) medium for 48 h at 37 °C under agitation. The culture was then washed twice in PBS, and a yeast suspension of 10^9^ CFU/mL in 200 µL of PBS or control medium (PBS) was administered daily to mice by intragastric gavage for 7 days before antibiotic treatment.

*Escherichia coli* MG1655 (ATCC, Molsheim, France) and *Enterococcus faecalis* and *Enterobacter hormaechei* isolated from feces of an antibiotic-treated mouse were also used in the study. The bacteria were grown on BHI medium for 24 h at 37 °C under agitation. The culture was then washed twice in PBS, and a bacterial suspension of 10^9^ CFU/mL in 200 µL of PBS/glycerol or control medium (PBS/glycerol) was stored in aliquots at −80 °C. This ready-to-use bacterial suspension was used for intragastric gavage for 10 days.

### Antibiotic treatments

Amoxicillin-clavulanic acid (150 mg/kg; Sandoz), ampicillin (200 mg/kg; Euromedex), colistin (600 μg/gavage; Sigma‒Aldrich), metronidazole (200 mg/kg; Toku-e), neomycin (200 mg/kg; Euromedex), vancomycin (100 mg/kg; Mylan) or a broad-spectrum antibiotic cocktail containing a mix of ampicillin (200 mg/kg; Euromedex), metronidazole (200 mg/kg; Toku-e), neomycin (200 mg/kg; Euromedex), and vancomycin (100 mg/kg; Mylan) were resuspended in NaCl as it is used in human intravenous treatments and administered to mice daily by intragastric gavage for 10 days.

### Tissues and samples

Mice were euthanized by cervical dislocation. The colon was flushed and frozen for further RNA extraction and biochemical measurements (myeloperoxidase activity level). Stomach, ileal, and cecal contents were collected and frozen for microbiota analysis. Fecal samples were collected and frozen for gut microbiota analysis and fecal lipocalin-2 level measurements. All samples were stored at −80 °C until use.

### Quantification of fecal lipocalin-2 (LCN2) levels

LCN2 quantification is used as a fecal biomarker for intestinal inflamation [18]. Frozen fecal samples were weighed and suspended in cold PBS. Samples were then agitated on a Precellys (Bertin Corp., France) for 40 s at 5000 rpm using 4.5-mm glass beads to obtain a homogenous fecal suspension. Samples were then centrifuged for 5 min at 10,000 *g* (4 °C), and clear supernatants were collected and stored at −20 °C until analysis. LCN2 levels were estimated using a DuoSet murine LCN2 ELISA kit (R&D Systems, Minneapolis, USA) according to the manufacturer’s instructions and expressed as pg/mg of stool.

### RNA extraction and gene expression analysis using quantitative real-time PCR

Total RNA was isolated from colon samples using an RNeasy Mini Kit (Qiagen, Hilden, Germany), including a DNAse treatment step, according to the manufacturer’s instructions. The quality and concentration of RNA were checked using a NanoDrop apparatus (Thermo Fisher Scientific, USA). Quantitative real-time PCR was performed using a LunaScript RT SuperMix Kit (New England Biolabs, Massachusetts, USA) followed by qPCR using a Luna^®^ Universal qPCR Master Mix (New England Biolabs, Massachusetts, USA) in a StepOnePlus apparatus (Applied Biosystems, Foster City, CA, USA) with specific mouse oligonucleotides. Amplification was initiated with an enzyme activation step at 95 °C for 10 min, followed by 40 cycles consisting of a 15-s denaturation step at 95 °C and a 60-s annealing step at 60 °C and a melting curve consisting of a temperature increase from 60 to 95 °C with a fluorescence analysis every 0.3 s. The primer sequences of the amplified targets are listed in Supplemental Table 1. We used the 2^−ΔΔCt^ quantification method with mouse GAPDH as a control.

### Myeloperoxidase (MPO) activity level

Abnormally high levels of MPO indicate significant or excessive neutrophil activation and is therefore used as a marker for inflammation [19]. To measure MPO activity, a 1-cm section of colon tissue was weighed and homogenized with Precellys (Bertin Corp., France) in 300 μL of a 0.5% hexadecyltrimethyl-ammonium bromide (HTAB, Sigma‒Aldrich) solution in 50-mM potassium phosphate buffer (PPB, pH 6.0); 0.35–0.40 mg of 1.4- and 2.8-mm ceramic beads (Ozyme, France) were added. Each sample was then vortexed for 10 s, centrifuged at 13,000 *g* and 4 °C for 10 min, and then transferred to a 96-well plate. To assay MPO activity, 50 μL of each aliquot was mixed with 200 μL of 50 mM PPB (pH 6.0) containing 0.167 mg/mL o-dianisidine-dihydrochloride (Sigma‒Aldrich, France) and 0.0005% hydrogen peroxide (H_2_O_2_, Sigma‒Aldrich). The colorimetric reaction was measured by reading the absorbance at 405 nm with a spectrophotometer (Infinite M200, Tecan, Switzerland) at two time points: immediately and after 1 h. MPO activity was characterized by comparison with a standard (MPO activity of human polymorphonuclear leukocytes, Merck Chemicals, Germany) and then expressed in units/mg of tissue. One activity unit represents the conversion of 1 μM H_2_O_2_ to water in 1 min at room temperature.

### Stomach, ileal, cecal, and fecal DNA extraction

Stomach, ileal, cecal, and fecal total DNA were extracted from weighed content samples as previously described [20], with modifications. After nucleic acid precipitation with isopropanol, DNA suspensions were incubated overnight at 4 °C and centrifuged at 20,000 × g for 30 min. The supernatants were transferred to a new tube containing 2 μL of RNase (RNase A, 10 mg/ml; EN0531; Fermentas, Villebon sur Yvette, France) and incubated at 37 °C for 30 min. Nucleic acids were precipitated by the addition of 1 ml of absolute ethanol and 50 μl of 3-M sodium acetate and centrifuged at 20,000 × g for 10 min. The DNA pellets were washed with 70% ethanol 3 times and dried and resuspended in 100 μl of Tris-EDTA (TE) buffer (10 mM Tris-HCl, 1 mM EDTA, adjusted pH 8).

DNA extraction from infant fecal samples was done as previously described [15, 16].

The DNA suspensions were stored at −20 °C for real-time qPCR analysis of the 16S rDNA or ITS2 sequences.

### Fungal and bacterial quantification via quantitative PCR (qPCR)

Content-extracted DNA was subjected to qPCR by using a Luna^®^ Universal qPCR Master Mix (New England Biolabs, MA, USA) for quantification of all fungal sequences or by using Luna^®^ Universal Probe qPCR Master Mix (New England Biolabs, MA, USA) for quantification of all bacterial sequences. For all fungal quantification, amplification was initiated with an enzyme activation step at 95 °C for 5 min, followed by 45 cycles consisting of a 15-s denaturation step at 94 °C, a 30-s annealing step at 55 °C, and a 30-s elongation step at 72 °C. This was followed by a single step of 5 min at 72 °C step and a melting curve step consisting of a temperature increase from 60 to 95 °C with a fluorescence analysis every 0.3 s. For all bacterial quantification, amplification was initiated with an enzyme activation step at 50 °C for 2 min and 95 °C for 10 min, followed by 40 cycles consisting of a 15-s denaturation step at 95 °C and a 60-s annealing/extension step at 60 °C. For *Enterobacteriaceae* and *Enterococcaceae* quantification, fecal-extracted DNA was subjected to qPCR by using a Luna^®^ Universal qPCR Master Mix (New England Biolabs, MA, USA), and amplification was initiated with an enzyme activation step at 95 °C for 20 s, followed by 40 cycles consisting of a 3 s denaturation step at 95 °C, a 30-s annealing step at 60 °C, and a melting curve step consisting of a temperature increase from 60 to 95 °C with a fluorescence analysis every 0.3 s. The probes and primers for the bacterial and fungal genes are listed in Supplemental Table 1. We used the 2^−ΔΔCt^ quantification method with feces weight and calibrated the assay to the control group.

### 16S DNA gene and ITS2 sequencing

Bacterial diversity was determined for each sample by targeting a portion of the ribosomal genes. PCR was performed to prepare amplicons using V3-V4 oligonucleotides (PCR1F_460: 5′ CTTTCCCTACACGACGCTCTTCCGATCTACGGRAGGCAGCAG 3′, PCR1R_460: 5′ GGAGTTCAGACGTGTGCTCTTCCGATCTTACCAGGGTATCTAATCCT 3′). Amplicon quality was verified by gel electrophoresis, and they were sent to the @BRIDGe platform for the sequencing protocol on an Illumina MiSeq (Illumina, San Diego, CA, USA).

A similar approach was used for fungal microbiota using the primers targeting the internal transcribed spacer 2 (ITS2) of the nuclear ribosomal genes in fungi (sense) 5′-GTGARTCATCGAATCTTT-3′ and (antisense) 5′-GATATGCTTAAGTTCAGCGGGT-3′ and the optimized and standardized ITS2-amplicon-library preparation protocol (Metabiote, GenoScreen).

### 16S and ITS2 sequence analysis

The 16S sequences were demultiplexed and quality filtered using the QIIME version 2.1.0 software package [21]. The sequences were then assigned to OTUs using the UCLUST algorithm [22] with a 97% pairwise identity threshold and classified taxonomically using the SILVA reference database (version 13.8) for bacteria [23]. For the ITS sequences, data were processed using the FROGS pipeline [24] for sequence quality control, and filtering and affiliation of taxa were performed with the UNITE ITS database (version 8_2) [25] using the FROGS guidelines for ITS data (http://frogs.toulouse.inra.fr/). Rarefaction analysis was performed and used to compare the relative abundance of OTUs across samples. Alpha diversity was estimated using the Shannon diversity index or the number of observed species. Beta diversity was measured using the Jaccard distance matrix and was used to build principal coordinates analysis (PCoA) plots. The linear discriminant analysis (LDA) effect size (LEfSe) algorithm was used to identify taxa that were specific to treatment [26]. In deposition of the raw sequence data in the SRA database from the NCBI, the accession numbers are the following: PRJNA861943.

### Mixed culture of bacteria and fungi

Bacteria isolated from feces of an antibiotic-treated mouse were cultivated with specific fungi. Briefly, feces from a mouse that received amoxicillin-clavulanic acid (150 mg/kg; Sandoz) for 10 days were grown on nutrient-rich BHI medium under aerobic conditions for 24 h at 37 °C. Bacteria were then isolated, purified, and identified by 16S PCR and sent to Eurofins Scientific (Eurofins, Nantes, France). In addition, *E. hormaechei* and *E. faecalis* isolated from mouse feces and *E. coli* MG1655 (ATCC, Molsheim, France) were also used in the study and grown on BHI in aerobic conditions for 24 h at 37 °C. The fungi *Saccharomyces cerevisiae* (MYCOTQ 1146 [4]) and *C. albicans* SC5314 (ATCC, Molsheim, France) were grown on YEPD medium under aerobic conditions for 24 h at 37 °C. Mixed cultures with fungi and bacteria (alive or heat-killed) or bacterial filtrated supernatant (0.2 µm) were grown at 37 °C for 24 h (ratio 1/1). Moreover, mixed cultures of *S. cerevisiae* with several filtrated supernatants (supernatant of 24-h mixed cultures of fungi and bacteria; supernatant of 24-h cultures of bacteria in YEPD, BHI, or YEPD/BHI) were performed. Additional mixed cultures included *S. cerevisiae* and antibiotic feces diluted in PBS (100 µL/mg of feces) and filtrated. For quantification, mixed cultures were plated either on YEPD agar plates supplemented with ampicillin (100 mg/mL; Euromedex), penicillin/streptomycin (10,000 units/mL penicillin and 10 mg/mL streptomycin, 1 mg/mL; Sigma‒Aldrich), or BHI agar plates supplemented with amphotericin B (100 µg/mL, Sigma‒Aldrich) and incubated at 37 °C for 24 h. Fungi and bacteria were then counted, and the absolute quantities of microorganisms were determined according to the corresponding dilutions.

### Statistical analysis

GraphPad Prism version 7 (San Diego, CA, USA) was used for all analyses and preparation of graphs. For all data displayed in graphs, the results are expressed as the mean ± SEM (*n* = 5 to 16 per group). The D’Agostino and Pearson test of normality was applied to all data sets, and in cases where the data did not demonstrate a normal distribution, nonparametric tests were used to analyze statistical differences. For comparisons between two groups, Student’s *t*-test for unpaired data or nonparametric Mann–Whitney test was used. For comparisons between more than two groups, one-way analysis of variance (ANOVA) and post hoc Tukey test or nonparametric Kruskal–Wallis test followed by a post hoc Dunn’s test was used. For all statistical tests, differences with a *p*-value less than 0.05 were considered statistically significant: *p < 0.05, **p < 0.01, and ***p < 0.001.

## Results

### Amoxicillin/clavulanic acid administration decreases the intestinal fungal population in both human and mouse models

As amoxicillin-acid clavulanic (Amox) is an antibiotic very commonly used in human, we used it to evaluate the effect of its daily administration on fungal gut microbiota in conventional mice. Antibiotics had a strong impact on bacterial quantity, characterized by a significant load decreased in the stomach, ileum, cecum, and feces compared to the control mice (Supp. Fig. 1A). Interestingly, the fungal population quantity again showed a significant loss in all compartments, especially in the ileum, the cecum, and the feces, after 10 days of daily gavage of Amox compared to the control (Supp. Fig. 1A).

**Fig. 1:**
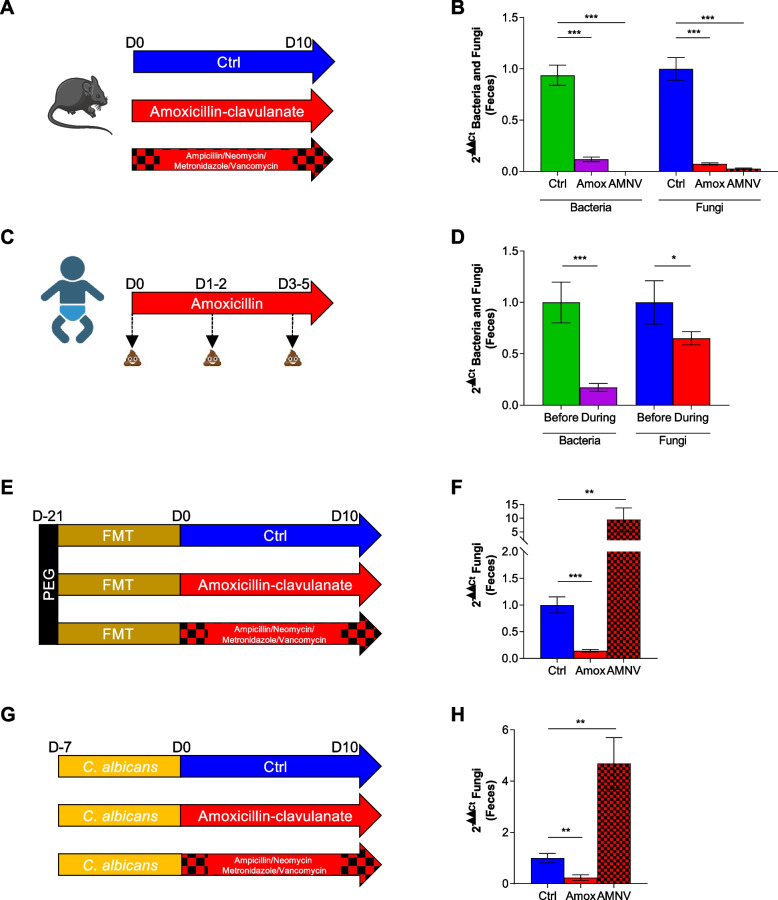
Different actions of antibiotics on fungal populations after treatment with amoxicillin/clavulanic acid or cocktail of ampicillin/metronidazole/neomycin/vancomycin in conventional, human microbiota-associated, or mice colonized by fungus. **A**–**B** Conventional mice treated with NaCl (Ctrl), amoxicillin/clavulanic acid (Amox), or a broad-spectrum antibiotic cocktail containing a mix of ampicillin, metronidazole, neomycin, and vancomycin (AMNV). Ctrl *n* = 24, Amox *n* = 32, AMNV *n* = 8, experiment was done 3 times. **C**–**D** Antibiotic-naïve infants treated with amoxicillin due to an infection with respiratory syncytial virus (RSV). Before *n* = 7, during *n* = 7. **E**–**F** Human microbiota-associated mice treated with Ctrl, Amox, or AMNV. Ctrl *n* = 16, Amox *n* = 24, AMNV *n* = 8, experiment was done 2 times. **G**–**H** Conventional mice colonized by *C. albicans* treated with Ctrl, Amox, or AMNV. Ctrl *n* = 13, Amox *n* = 16, AMNV *n* = 20, experiment was done 3 times. **A**, **C**, **E**, and **G** Experimental design for the administration of antibiotics in conventional mice (**A**), infants (**C**), human microbiota-associated mice (**E**), and mice colonized by fungus (**G**). **B** Bacterial and fungal quantity in feces after 10 days of antibiotics, determined by qPCR. **D** Bacterial and fungal quantity in feces after 1 to 5 days of amoxicillin treatment, determined by qPCR. **F**–**H** Fungal quantity in feces after 10 days of antibiotics, determined by qPCR. *p < 0.05, **p < 0.01, ***p < 0.001

For a better understanding of the antibiotic-related effects on the fungal population, we repeated the experiment with a broad-spectrum antibiotic treatment of ampicillin, metronidazole, neomycin, and vancomycin (AMNV) (Fig. 1A), which has been reported to increase fungal populations [13]. We confirmed our first observations: antibiotic treatments affect the bacterial population but also strongly affect the fungal burden, which is characterized by a significant loss in the feces (Fig. 1B). With the aim of confirming these results in a human setup, we used samples originated from a previous study. In this study, the K-L. Kolho lab [15, 16] monitored the effect of antibiotics on early-life gut microbiota equilibrium in an infant cohort and demonstrated specific modifications of the bacterial and fungal microbiota diversity and composition, but no data were available on the modulation of the microbial load during such treatments. We selected from the initial cohort, longitudinal fecal samples from seven infants before and during the administration of amoxicillin and quantified the bacterial and fungal composition (Fig. 1C). We showed that amoxicillin, even alone, induced a decrease on bacterial quantity and also on fungal load as observed in mice (Fig. 1D).

To evaluate the effects of the specific antibacterial treatment in modulating the fungal microbiota in human feces, we used a human microbiota of a healthy adult for fecal microbiota transfer (FMT) in conventional mice to create human microbiota-associated (HMA) mice and administered Amox or AMNV for 10 days to these mice (Fig. 1E). We then compared their fungal microbiota to that of the untreated control mice. We observed an antibiotic-dependent response. While AMNV increased fungal levels in the feces, Amox had the opposite effect, characterized by a significant decrease in the fungal quantity (Fig. 1F). We also evaluated the effect of our antibiotics on conventional mice colonized by a well-known fungus, *Candida albicans* (Fig. 1G). Again, AMNV, but not Amox, increased fungal quantity (Fig. 1H). We then investigated which antibiotic could increase fungal quantity in AMNV administration by using them independently or in double combinations in mice colonized by *C. albicans* (Supp. Fig. 2)*.* Depending on the drug used, the fungal population seemed affected, either decreased or increased compared to the control mice. However, it was only the addition of the four antibiotics together that showed a significant effect.

We showed that Amox administration triggered a significant loss in bacterial and fungal populations in infant feces and in several compartments in conventional, HMA, and *C. albicans*-colonized mice. As a possible explanation for this phenotype, we investigated the host immune response after Amox treatment, looking for a possible indirect effect of Amox on the host that would elicit this modification of the fungal population. We investigated whether Amox exacerbates intestinal inflammation and thus impacts the microbial equilibrium. We measured the lipocalin-2 (LCN2) concentration in the mouse feces after 10 days of Amox but detected very low levels of lipocalin, far from any sign of clear inflammation (Supp. Fig. 3A). The modification of the microbiota did not seem to be the consequence of modification of neutrophil recruitment since myeloperoxidase (MPO) activity quantification in feces or quantification of related markers such as Lys1, Nos2, or Cxcl-2 indicated no significant modulation (Supp. Fig. 3B). We also measured antimicrobial peptide expression in the colon following Reg3β, S100A8, and S100A9 (Supp. Fig. 3C) and only noted a significant but small increase with S100A8. Globally, no strong signals are observed in the host response but a tendency of a slight modification. Additionally, the decrease in the fungal load in the gut after Amox treatment was associated with a tendency toward decreased IL-17A expression, which is expected since IL-17 is notably associated with fungal detection (Supp. Fig. 3D).

To dismiss a direct effect of Amox on fungal growth, drop tests were performed on Petri dishes supplemented with Amox, and no impact on *C. albicans*, *C. tropicalis*, or *Saccharomyces cerevisiae* growth was detected (data not shown).

### Amoxicillin/clavulanate treatment alters the bacterial and fungal composition

**Fig. 2:**
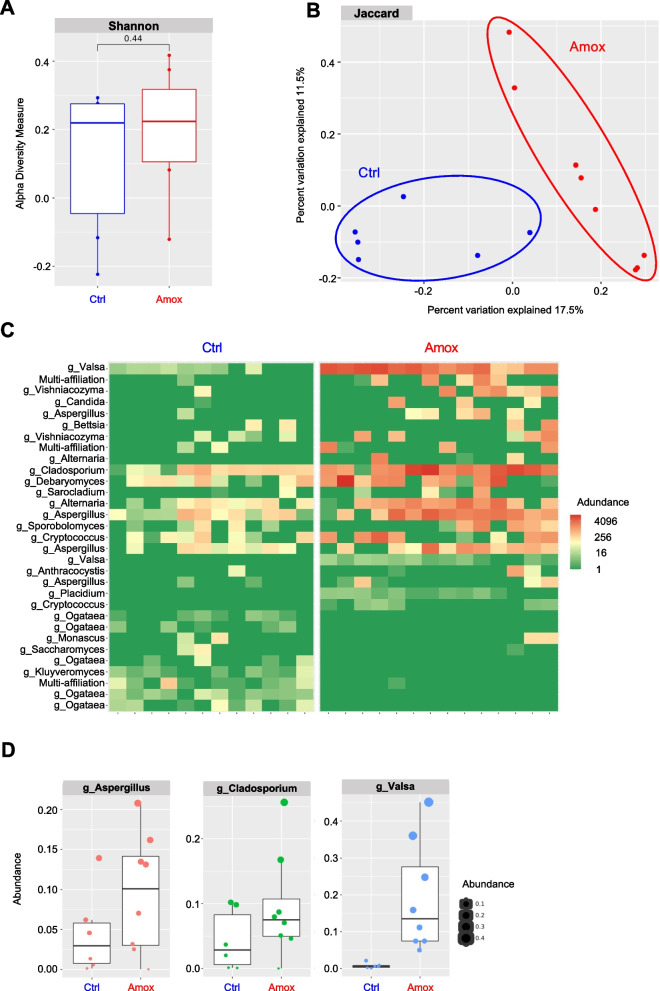
Amoxicillin/clavulanate acid treatment alters the fungal microbiota. **A**–**D** Conventional mice treated with NaCl (Ctrl) or amoxicillin/clavulanic acid (Amox). Ctrl *n* = 6–12, Amox *n* = 8–14. A. Shannon index describing the alpha diversity of the fungal microbiota (ITS) in the fecal microbiota after 10 days of antibiotic treatment. **B** Beta diversity. Principal coordinate analysis of Jaccard distances with each sample colored according to the treatment. **C** Differential analysis by DeSeq2. **D** Relative abundance (% reads) of families specifically increased or decreased by antibiotic treatment

To investigate the possible mechanism explaining this decrease, we explored the effects of Amox on both fungal and bacterial intestinal microbiota in mice. First, we surveyed the effects on the fungal population (Fig. 2). Fecal ITS2 sequence analysis showed a trend of alpha diversity increase (Shannon index) after Amox treatment compared to the control treatment (Fig. 2A), as previously described [16]. A beta-diversity analysis (Jaccard index) confirmed the effect of Amox on the global fungal microbiota; indeed, the two groups “Amox” versus “Ctrl” formed two significantly separated clusters (*p*-value 0.001) (Fig. 2B). DesEq2 differential analysis showed significant differences in several taxonomic ranks (Fig. 2C), including an increase in the *Aspergillus*, *Cladosporium*, and *Valsa* genera between Amox and nontreated mice (Fig. 2D).

**Fig. 3:**
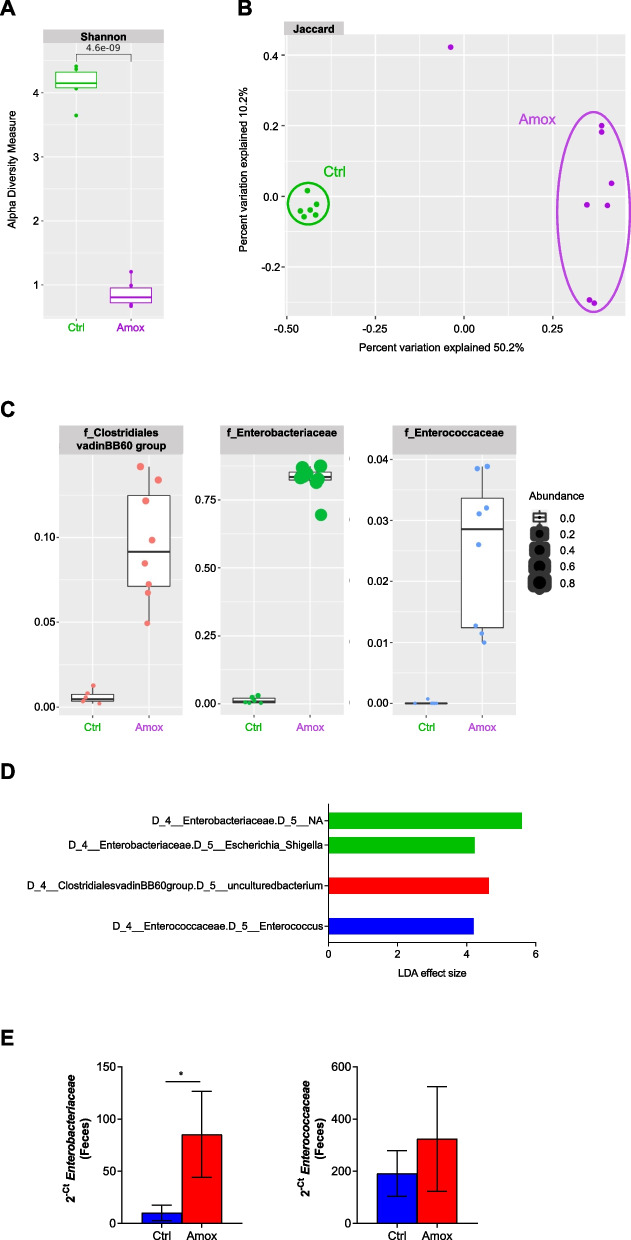
Amoxicillin/clavulanate acid treatment alters the bacterial microbiota. **A**–**D** Conventional mice treated with NaCl (Ctrl) or amoxicillin/clavulanic acid (Amox). Ctrl *n* = 6, Amox *n* = 8. **A** Shannon index, describing the alpha diversity of the bacterial microbiota (16S) in the fecal microbiota after 10 days of antibiotic treatment. **B** Beta diversity. Principal coordinate analysis of Jaccard distances with each sample colored according to the treatment. **C** Taxa with the largest differences (*LDA* > 2) in abundance by linear discriminant analysis (LEfSe) (*LDA* > 2). **D** Relative abundance (% reads) of families specifically increased by antibiotic treatment. **E** Antibiotic-naïve infants treated with amoxicillin due to an infection with respiratory syncytial virus (RSV). Before *n* = 7, during *n* = 7. **E** *Enterobacteriaceae* and *Enterococcaceae* quantity in feces after 1 to 5 days of Amox, determined by qPCR. *p < 0.05

We confirmed using fecal 16S RNA amplicon sequencing that the antibiotic treatment significantly reduced the bacterial alpha-diversity (Shannon index) compared to the control group (Fig. 3A) and a specific dysbiosis illustrated by the clear clustering of the treated and untreated groups (Jaccard index, *p*-value 0.001) (Fig. 3B). Amox treatment induced global changes in the bacterial microbiota with a strong decrease in many bacterial families, but these ecosystem modifications also favored the development of several other families. Differential analysis using the LeFSe tool showed that *Enterobacteriaceae*, *Clostridiales vadinBB60 group*, and *Enterococcaceae* were significantly enhanced after 10 days of Amox treatment with strong modification of their abundances in these environmental conditions (Fig. 3 C–D). Our collaborators [15] already showed an increase in the Enterobacteriaceae family in infants treated with different antibiotics (amoxicillin and macrolide) but observed a decrease in the *Clostridiaceae* and *Enterococcaceae* families with Amox, and we confirmed these observations by qPCR (Fig. 3E). Unfortunately, we were not able to quantify the *Clostridiales vadin BB60 group* family, as it is a specific group largely unclassified and uncharacterized with no primers currently available.

### Enterobacteriaceae have an impact on the fungal population

**Fig. 4:**
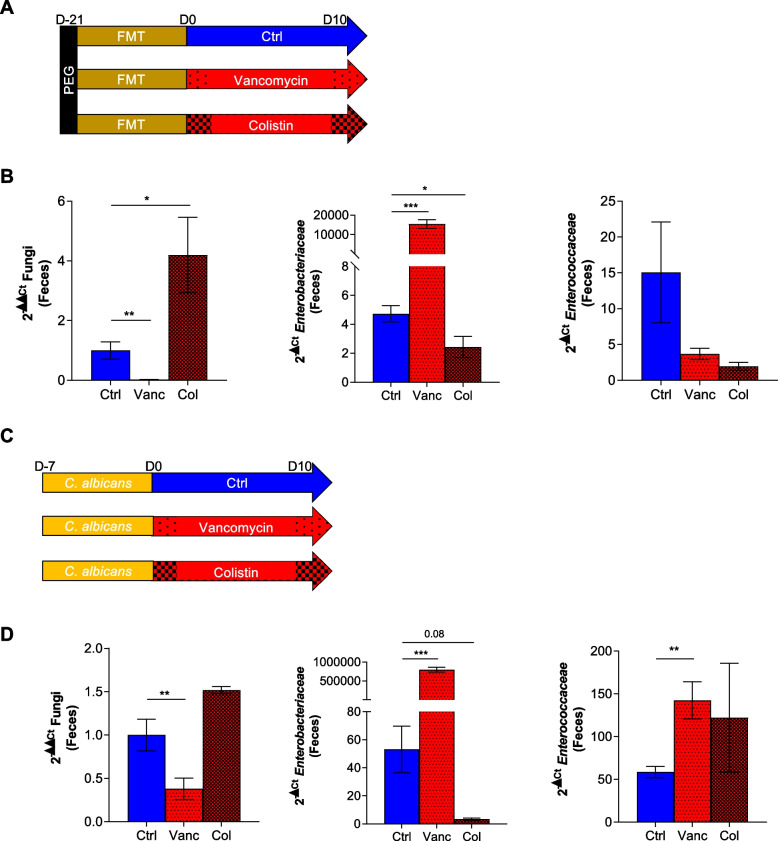
Different actions of antibiotics targeting the Enterobacteriaceae family on fungal populations after treatment with vancomycin or colistin in human microbiota-associated mice colonized by a fungus. **A**–**B** Human microbiota-associated mice treated with NaCl (Ctrl), vancomycin (Vanc), or colistin (Col). Ctrl *n* = 8, Vanco *n* = 8, Col *n* = 8, experiment was done 1 time. **C**–**D** Conventional mice colonized by *C. albicans* treated with Ctrl, Vanco, or Col. Ctrl *n* = 13, Vanco *n* = 16, Col *n* = 5, experiment was done 2 times. **A** and **C** Experimental design for the administration of antibiotics in human microbiota-associated mice (**A**) and mice colonized by a fungus (**C**). **B** and **D** Fungal, *Enterobacteriaceae*, and *Enterococcaceae* quantity in feces after 10 days of antibiotic treatment, determined by qPCR. *p < 0.05, **p < 0.01, ***p < 0.001

As the *Enterobacteriaceae* family was strongly increased in Amox infants and mice, we wondered if these bacteria could have a negative impact on fungal growth. For a better understanding, we conducted two in vivo experiments with antibiotics that have two different effects on *Enterobacteriaceae*: vancomycin (Vanc), which is known to target gram-positive bacteria, consequently triggering a bloom of *Enterobacteriaceae*, and colistin (Col), which targets *Enterobacteriaceae*, dramatically reducing their abundance [27]. As previously done, we performed FMT on mice with feces of a human healthy subject and administered Vanc or Col for 10 days (Fig. 4A) to evaluate the effects on a fungal microbiota closer to the human microbiota. We observed that Vanc induced a decrease in fungal abundance concomitant with a significant increase in *Enterobacteriaceae* levels, as expected (Fig. 4B). On the other hand, Col induced an increase in fungal abundance, while the levels of *Enterobacteriaceae* decreased strongly (Fig. 4B). Moreover, we also quantified the *Enterococcaceae* abundance levels in those mice, as this family was also increased by Amox treatment (Fig. 3 C–D), and we did not observe any difference between the groups (Fig. 4B). We confirmed these results using conventional mice colonized by *C. albicans* by oral gavages (Fig. 4 C–D).

### The effects of Enterobacteriaceae on fungal cells are complex and could be a combination of different phenomena

**Fig. 5:**
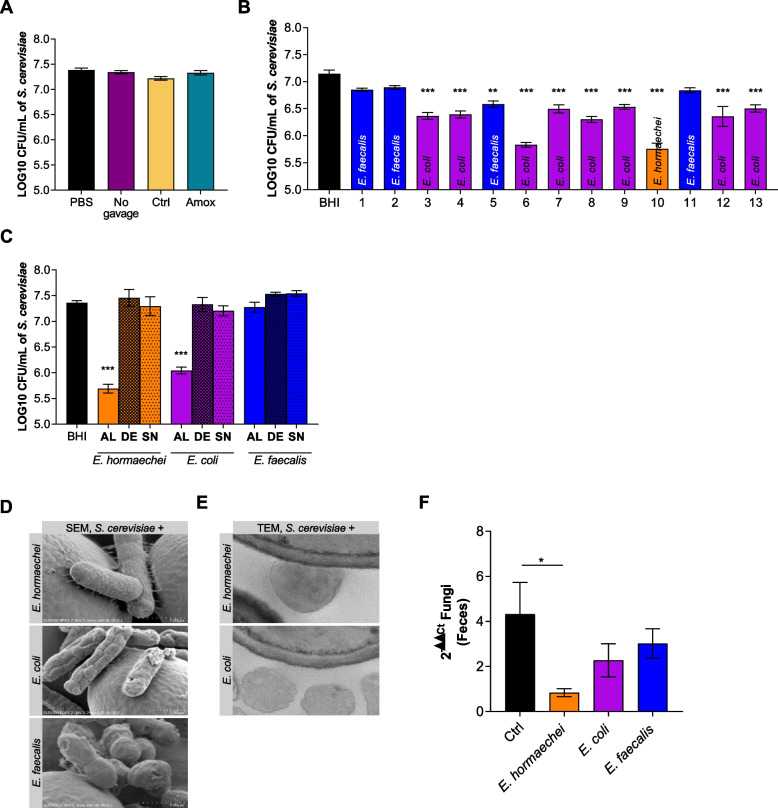
Effects of *Enterobacteriaceae* and *Enterococcaceae* on fungi. **A** Fungal quantity of mixed cultures of *S. cerevisiae* and feces from mice treated with no gavage, NaCl (Ctrl) or amoxicillin/clavulanic acid (Amox) diluted in PBS, expressed in CFU. Experiment was done 1 time. **B** Fungal quantity of mixed cultures of *S. cerevisiae* and bacteria isolated from feces of an antibiotic mouse, expressed in CFU. For statistical comparisons, (*) indicates versus BHI, experiment was done 3 times. **C** Fungal quantity of mixed cultures of *S. cerevisiae* and *Enterobacter hormaechei* either alive (AL) or dead (DE) or the corresponding bacterial supernatant (SN), *Escherichia coli* AL, DE, or SN, or *Enterococcus faecalis* AL, DE, or SN, expressed in CFU. For statistical comparisons, (*) indicates versus BHI, experiment was done 6 times. **D** Scanning electron microscopy (SEM) analyses of mixed cultures of *S. cerevisiae* and *E. hormaechei* (upper panel), *E. coli* (middle panel), or *E. faecalis* (lower panel) (1 µm). **E** Transmission electron microscopy (TEM) analyses of mixed cultures of *S. cerevisiae* and *E. hormaechei* (upper panel) or *E. coli* (lower panel). **F** Human microbiota-associated mice treated with PBS/glycerol (Ctrl), *E. hormaechei*, *E. coli*, or *E. faecalis*. Ctrl *n* = 15, *E. hormaechei n* = 15, *E. coli n* = 15, *E. faecalis n* = 15, experiment was done 2 times. **F** Fungal quantity in feces after 10 days of bacterial treatment, determined by qPCR. *p < 0.05, **p < 0.01, ***p < 0.001

Microbial composition seemed to have an effect on fungal growth. As an initial screening for effectors of this inhibitory effect, we tested whether metabolites potentially present in the feces would have an impact on fungal growth in vitro. To investigate the role of in vivo produced metabolites, we added the aqueous phase (fecal water) of feces from mice not treated, treated with vehicle (Ctrl, NaCl), or treated with Amox to *S. cerevisiae* liquid culture. We used *S. cerevisiae* because it is largely present in the human gut and because *Saccharomyces* genus was also the genus impacted by Amox treatment in infants (Fig. 2D) [16]. Neither of these fecal waters had any positive nor negative impact on fungal growth (Fig. 5A).

Since the metabolites remaining in the feces did not seem to be involved in the decrease in fungal quantity in Amox mice, we explored the potential effect of bacterial strains themselves. Thus, we initiated the isolation of aerobic bacteria from the feces of mice treated for 10 days with Amox: isolated bacteria were then coincubated with *S. cerevisiae*. Nine of the 13 isolated bacteria had a significant negative impact on *S. cerevisiae* growth (*p* < 0.001), and after identification, all of them were from the Enterobacteriaceae family (Fig. 5B).

Based on these results, we evaluated the effects of three bacteria, *Enterobacter hormaechei* and *Enterococcus faecalis* isolated from our antibiotic mice and *Escherichia coli* MG1655, a well-described *E. coli* strain, using alive or dead bacteria or their culture supernatant. Neither dead bacteria nor their supernatant had any negative impact on *S. cerevisiae* growth (Fig. 5C). We also evaluated the impact of these three bacteria on another yeast, *C. albicans*, and noted that *Enterobacteriaceae* and particularly *E. hormaechei* had a significant negative impact on fungal growth (Supp. Fig. 4A). Dilution of the bacterial load demonstrated that the bacteria must be in sufficient concentration to have an effect on the fungus (Supp. Fig. 4B).

To further investigate how *Enterobacteriaceae* could affect fungal growth, we designed different experiments:

• Firstly, the effect of the coculture with *S. cerevisiae* on bacterial growth was assessed over 10 h, followed by a growth curve using CFU counts. We did not observe any modification of either *E. hormaechei* or *E. coli* growth in the presence of *S. cerevisiae* (Supp. Fig. 4C, middle and lower panels).
• Secondly, we compared the growth of *S. cerevisiae* alone or with the two bacteria over a time course. While the three curves were superimposed during the first 6 h, they began to diverge after that, with a slower growth rate of both cocultures compared to the *S. cerevisiae* monoculture (Supp. Fig. 4C, upper panel). However, when growing *S. cerevisiae* in a medium where both bacteria and fungi had grown for 24 h, we did not see any effect on *S. cerevisiae* growth (Supp. Fig. 4D).
• Thirdly, we added the supernatant of a 24-h culture of *E. hormaechei*, *E. coli*, or *E. faecalis* in different media (YEPD, BHI, or YEPD/BHI vol/vol) to a fresh culture of *S. cerevisiae*. Under these conditions, we noted a slight effect of spent media on the growth of *S. cerevisiae*, but it was not as potent as what was observed in coculture (Supp. Fig. 4E), suggesting that the depletion of the medium in some nutrients could be partially responsible for the fungal growth decrease.

To understand whether specific interactions occurred between these bacterial and fungal cells, we used scanning (SEM) and transmission electron microscopy (TEM) observations. While all three bacteria were observed in contact with the yeast cells, specific features were visible in the case of the coculture of *E. hormaechei* and *S. cerevisiae* cells. SEM images showed the presence of numerous pili-like structures linking *E. hormaechei* and *S. cerevisiae* cells (Fig. 5D), and no similar structures were observed with the two other bacteria. Nevertheless, these pili-like structures seemed to be an intrinsic property of *E. hormaechei* (Supp. Fig. 5A) and were not developed in response to the presence of the fungus. We were not able to obtain TEM images with both microorganisms for all coincubations, and no images of *E. faecalis* close to yeast cells were found. However, for the two other bacteria, TEM showed intimate contact between *E. hormaechei* and *S. cerevisiae* cells but not with *E. coli* cells (Fig. 5E). Concerning the fungal cell ultrastructure in the presence of the three bacteria, we observed a disorganization in the organelles visible in the cytoplasm of yeast in the presence of *E. hormaechei* but not in the presence of the two other bacteria (Supp. Fig. 5B).

Finally, to further elucidate these bacterial effects on the fungal microbiota in general, we tested whether we could recapitulate the effect of antibiotic treatment with the use of the bacteria identified in our study in vivo. We administered the three bacteria separately to HMA mice for 10 days. The two *Enterobacteriaceae* bacteria were able to reduce the intestinal fungal load in vivo, but only *E. hormaechei* had a significant effect (Fig. 5F).

Overall, these results suggest that the bacterial dysbiosis induced by amoxicillin/clavulanic acid administration decreases the fungal population. It is likely that the Enterobacteriaceae family, which is increased by Amox treatment, is at least in part involved in this negative modulation through various mechanisms, such as competition for specific nutrients and cell-to-cell adhesion, which would potentialize the competition.

## Discussion

The aim of this article was to study the effects of antibiotic treatment on the fungal microbiota, since very little has been done on this subject this far. This is particularly important because of the increasing amount of published data suggesting a role of the fungal community in the microbiota equilibrium and in host health [7, 8].

It is widely considered that during antibiotic treatment, bacterial niches newly released by the treatment become suitable for the expansion of fungi, as it is observed with an increase in the fungal load [10, 11, 13]. Moreover, after stopping antibiotic treatment, bacteria normally return to their normal levels and niches, while fungi drop back to their baseline levels [13].

Amoxicillin-clavulanic acid (Amox) is one of the most commonly used antibiotics in humans to treat a large scale of infections with rather high efficacy [28]. As such, we chose to study the effect of Amox on the host and the balance of the bacterial and fungal microbiota. In vivo experiments on mice showed surprising differences in the effect of antibacterial treatment on the fungal community, demonstrating that depending on the antibacterial molecule used, the effect on the global fungal population could be completely opposite. Interestingly, Amox treatment, instead of promoting fungal overgrowth, resulted in a decrease in the fungal population global abundance. We demonstrated it using various in vivo mice model: conventionnal mice, mice orally gavaged with fungi, or mice after fecal microbiota transfer (FMT) with feces from a healthy human donor. As a partial confirmation of the effects of Amox on the fungal microbiota in human, we also used samples from an already described cohort collected in K-L Kolho’s laboratory [15]. Although the modification of the fungal microbiota diversity and composition has already been described, nothing has been reported on the fungal load after antibacterial treatments [16]. Using these samples, we were able to confirm a decrease in the fungal load after amoxicillin treatment alone. While these results cannot be compared completely with the Amox treatment and were obtained on a small cohort, they suggested that similar effects might occur in humans. In the future, gathering human samples before and after treatment with Amox would help confirming this hypothesis.

In an attempt to explain these unexpected observations, we tested diverse hypotheses: direct effect of the Amox on the fungal growth or an umbalanced host immune response; none of them was satisfactory. The last hypothesis was that there was an impact of the antibiotic on the bacterial community; hence, this dysbiosis would impact the fungal community. Using data from the 16S sequences, we identified three bacterial families with increased relative abundance during antibiotic therapy with Amox: *Enterobacteriaceae*, *Clostridiales vadinBB60 group*, and *Enterococcaceae*. A recent study following the effects of antibiotics on infant gut microbiota also showed partially comparable effects with a significant increase in the relative abundance of *Enterobacteriaceae* (but not the others) when treated with amoxicillin alone [15].

To study whether these bacterial families, which are resistant to Amox, could indeed have a negative impact on the fungal quantity, we isolated aerobic bacteria from the feces of mice treated with Amox for 10 days and screened them using coculture with a fungal strain from the *Saccharomyces* family specifically impacted by amoxicillin administration [16]. From this screen, we observed that bacteria from the Enterobacteriaceae family, unlike *Enterococcaceae*, decreased fungal growth suggesting that this particular family could affect the fungi. *Enterobacter hormaechei* was the most potent strain identified by this screening approach, with higher efficiency compared to *Escherichia coli* from our lab collection.

In an attempt to decipher the mechanisms underlying the deleterious effect of *E. hormaechei*, we used several experimental procedures with spent medium or dead bacteria that suggested that the bacteria needed to be alive but was not producing toxic metabolites.

Another way to test the relationship between two organisms in coculture is to compare growth curves of single growth of each organism to that under the coculture of both. If the coculture growth rate is reduced when the single culture reaches its stationary phase, it is called the “Jameson effect” after Jameson’s observations in 1962 [29]. The Jameson effect is considered as a nonspecific competition for nutrient, but this effect has been described so far only between bacteria and not between fungi and bacteria [30, 31]. Our study suggested that at one point, the medium lacked the molecules necessary for the fungal cells to fully develop. Even if the stop of growth was not as quick as in other models, these results advocated that part of the effect could be due to the competition for nutrients, also known as the Jameson effect. However, since growth of *S. cerevisiae* in bacterial-spent medium was not affected, it was likely that yet unknown parameters were also at play in this growth reduction.

SEM and TEM observations were then conducted to identify possible physical interactions that may affect fungal growth. Even if no difference in the number of bacterial cells in contact with the yeast cells was observed, we showed that *E. hormaechei* and yeast cells were tightly linked together by fimbriae-like structures and showed intimate contact that was not present with the two other bacteria.

**Fig. 6:**
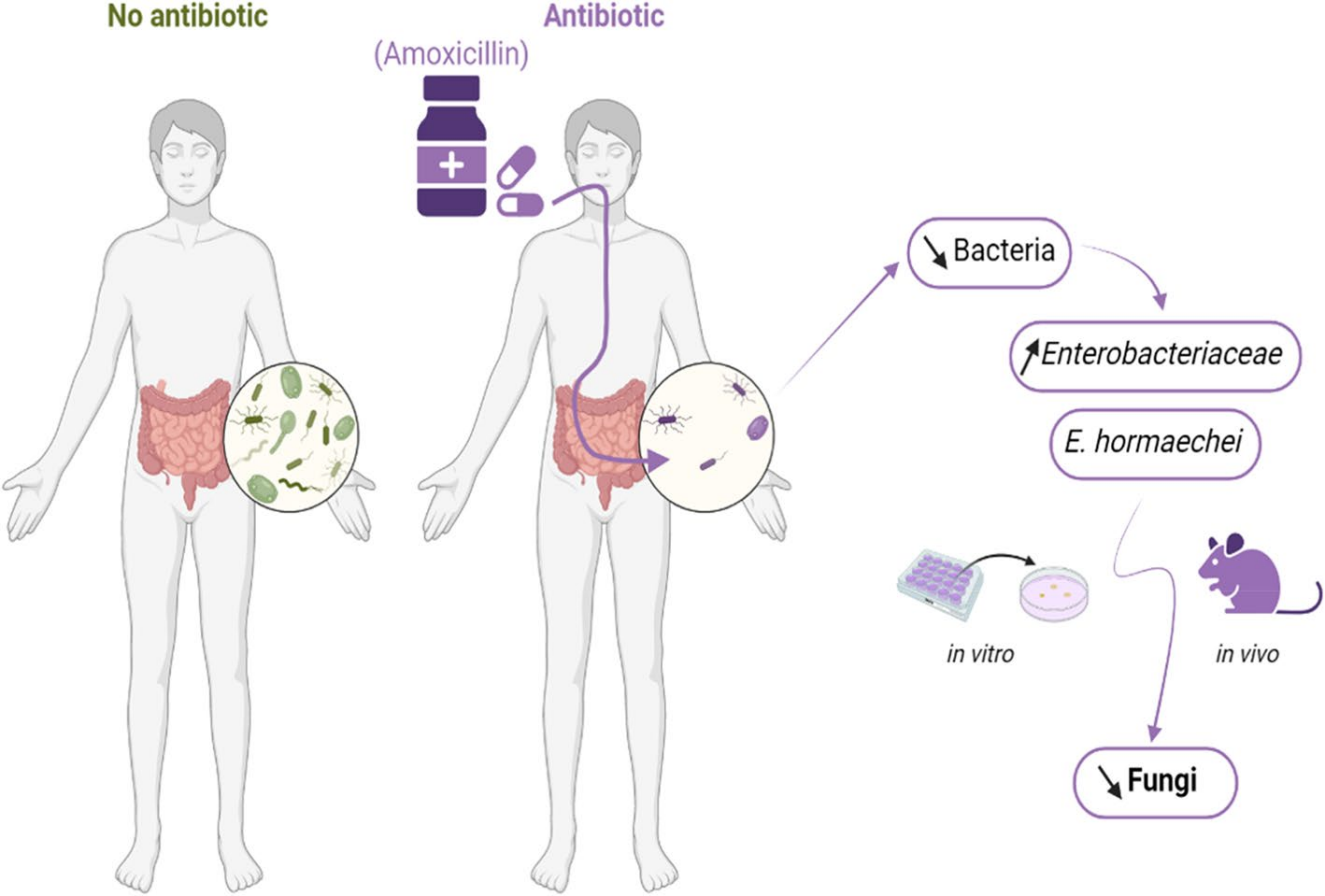
Graphical abstract of the present study

Interestingly, when provided to the mice by oral gavage, *E. hormaechei* was able to recapitulate the phenotype observed after Amox treatment with a significant reduction in the global fungal population, confirming the potential effect of this bacterium on the growth of the fungal cells in vivo (Fig. 6).

Studies on the interaction between bacteria and fungi in vivo are a very young area of research. The two microbiota have been first studied separately, but it is now evident that one cannot ignore the multiple interactions that are certainly occurring between these two types of organisms within these microbial ecosystems. Many reports exist describing the different effects of bacteria and fungi on each other, but they are mainly based on in vitro assays and interactions between one bacterial strain and one fungal strain [32]. Additionally, studies in many cases have focused on pathogenic microorganisms; thus, the literature lacks data exploring the interactions between commensal fungi and bacteria, events that occur on a daily basis within our microbiota [33].

However, studying these interactions in vivo is extremely complex, as the microbiota composition can greatly influence the experimental output. For instance, the effects in vivo of *Enterobacteriaceae* on the fungal community can go in various directions. We have shown in a previous study that *Enterobacteriaceae* were necessary in the gut for the development of pro- or anti-inflammatory effects in a model of colitis with two fungal strains: *C. albicans* and *S. boulardii* [34]. Hoarau and coworkers [35] have shown that *Serratia marcescens* and *E. coli*, two *Enterobacteriaceae*, positively correlate with *C. tropicalis* in Crohn’s disease patients, and they also demonstrated the presence of adhesion phenomena and the potential implication of fimbriae. However, it has been demonstrated in other studies that *Enterobacteriaceae* can be deleterious to fungi. For instance, in a recent study, the authors described the production by *E. coli* of a soluble fungicidal factor, a factor that can kill *C. albicans* in a magnesium-dependent fashion [36]. *Enterobacter* strains have also been implicated in different experiments showing inhibitory effects on fungal cells. Several *Enterobacter* stains have shown inhibitory effects on different fungal strains: Chernin L. et al. [37, 38] described the chitinolytic activity of secreted proteins of *Enterobacter agglomerans* that can inhibit the development of fungal plant pathogens or *E. agglomerans* production of pyrrolnitrin, an antibiotic molecule that has broad-spectrum antagonistic activity against fungal filamentous strains. Additionally, a recent publication describes *Enterobacter asburiae* and its production of volatile compounds that can reduce the growth of *Aspergillus flavus* and 7 other fungal plant pathogens [39].

However, there is no publication describing *E. hormaechei*-specific interactions with fungal strains to date. *E. hormaechei* was described in 1989 and is very close to *Enterobacter clocae*, and as such is part of the phylogenic “*E. clocae* complex”; *E. hormaechei* strains have been isolated from diverse human niches (wounds, urine, or blood) but are found in various other environments (soil, water, vertebrate host, vegetation, etc.) [40]. This group has been associated with various nosocomial infections, especially in neonates. *Enterobacter* are known to be motile for most species, aerobic, and with frequent resistance to beta-lactam antibiotics [41]. These data partially explain the increased presence of this strain in the Amox-treated samples and the observation of fimbriae on our microscopic pictures that might contribute to its mobility.

In our study, the deleterious effects on fungal growth seemed to be partially explained by metabolite/nutrient competition also described as the Jameson effect. However, in vitro assays suggest that this is not the only mechanism. The fact that *E. hormaechei* is developing a network of fimbriae can possibly explain a potentiation of the effect on fungal growth by a strong modification of the local conditions of growth or specific exchange of molecules that we were not able to isolate in vitro.

## Conclusion

Our work did not verify the paradigm stating that after any antibacterial treatment, the fungal microbiota will benefit from the release of the competition as we discovered an exception. Treatment with amoxicillin and clavulanic acid can have the opposite effect in human or in a mouse model through indirect impacts on the bacterial microbiota. However, our data are the results of in vivo experiments in mice or came from small human cohort and thus have to be taken with precaution until the collection of data from much larger cohorts. Nonetheless, these data help us better understand the gut microbiota equilibrium and might lead to new hypotheses to modify medical practices in some specific situations where the fungal microbiota can strongly influence the host health.

## Supplementary Information

**Additional file 1:** **Supplemental Table 1.** The primer sequences of the amplified targets

**Additional file 2:** **Supplemental Fig. 1.** Bacterial and fungal populations decrease after treatment with amoxicillin/clavulanic acid in conventional mice. (A) Conventional mice treated with NaCl (Ctrl) or amoxicillin/clavulanic acid (Amox). Ctrl *n* = 8, Amox *n* = 16, experiment was done 1 time. A. Bacterial and fungal quantity in several compartments after 10 days of antibiotic treatment, determined by qPCR. *p <0.05, **p <0.01, ***,*p*<0.001

**Additional file 3:** **Supplemental Fig. 2.** Different actions of antibiotics on fungal populations after treatment with ampicillin/metronidazole/neomycin/vancomycin in mice colonized by a fungus. (A) Conventional mice colonized with C. albicans treated with Ctrl, ampicillin (A), metronidazole (M), neomycin (N), vancomycin (V) or their mixture (AM, AN, AV, MN, MV, NV, AMNV). *n* = 5 per group, experiment was done 2 times. A. Fungal quantity in feces after 10 days of antibiotics, determined by qPCR. ***, *p*<0.001

**Additional file 4:** **Supplemental Fig. 3.** Host response after 10 days of treatment with amoxicillin/clavulanic acid in conventional mice. (A-D) Conventional mice treated with NaCl (Ctrl) or amoxicillin/clavulanic acid (Amox). Ctrl *n* = 8, Amox *n* = 16. A. Intestinal inflammation, expressed by lipocalin-2 levels in feces. B. Neutrophil recruitment, expressed by myeloperoxidase (MPO) activity in the colon (left panel) and markers in the colon (qPCR, right panel). C. Antimicrobial peptides expressed in the colon (qPCR). D. Antifungal immunity expressed in the colon (qPCR). *p <0.05

**Additional file 5:** **Supplemental Fig. 4.** Effect of Enterobacteriaceae and Enterococcaceae on fungi. A. Fungal quantity of mixed cultures of Candida albicans and Enterobacter hormaechei either alive (AL) or dead (DE) or the corresponding bacterial supernatant (SN), Escherichia coli AL, DE or SN or Enterococcus faecalis AL, DE or SN, expressed in CFU. For statistical comparisons, (*) indicates versus BHI, experiment was done 6 times. B. Fungal quantity of mixed cultures of S. cerevisiae and E. hormaechei (upper panel) or E. coli (lower panel) at decreasing concentrations of bacteria, expressed in CFU. For statistical comparisons, (*) indicates versus BHI, experiment was done 2 time. C. Fungal (upper panel) and bacterial (middle and lower panels) quantities of mixed cultures of S. cerevisiae and E. hormaechei or E. coli at several hours, expressed in CFU. For statistical comparisons, (*) indicates versus BHI, experiment was done 2 times. D. Fungal quantity of mixed cultures of S. cerevisiae and SN of 24 h mixed cultures of S. cerevisiae and E. hormaechei or E. coli, expressed in CFU. Experiment was done 2 times E. Fungal quantity of mixed cultures of S. cerevisiae and SN of 24 h cultures of E. hormaechei or E. coli in YEPD, BHI or YEPD/BHI v/v, expressed in CFU. For statistical comparisons, (*) indicates versus the appropriate control: YEPD, BHI or YEPD/BHI, experiment was done 2 times. *p <0.05, **p <0.01, ***p<0.001

**Additional file 6:** **Supplemental Fig. 5.** Scanning and transmission election microscopy analyses. A. Scanning electron microscopy (SEM) analyses of E. hormaechei (1 µm). E. Transmission electron microscopy (TEM) analyses of mixed cultures of S. cerevisiae and E. hormaechei (upper panel), E. coli (middle panel) or E. faecalis (lower panel).

## Data Availability

All data generated or analyzed during this study are included in this published article (and its supplementary information files). Deposition of the raw sequence data in the European Nucleotide Archive is as follows: https://www.ncbi.nlm.nih.gov/bioproject/PRJNA861943.

## Abbreviations

A: Ampicillin
AL: Alive
AMNV: Ampicillin, metronidazole, neomycin, and vancomycin
Amox: Amoxicillin-clavulanic acid
ANOVA: Analysis of variance
BHI: Brain-heart infusion
C. albicans: Candida albicans
C. tropicalis: Candida tropicalis
CFU: Colony-forming unit
Col: Colistin
Ctrl: NaCl
DE: Dead
E. coli: Escherichia coli
E. faecalis: Enterococcus faecalis
E. hormaechei: Enterobacter hormaechei
FMTFecal: microbiota transfer
HMA: Human microbiota associated
HTAB: Hexadecyltrimethylammonium bromide
IL-17A: Interleukin-17A
LCN2: Lipocalin-2
LDA: Linear discriminant analysis
LEfSe: LDA effect size
M: Metronidazole
MPO: Myeloperoxidase
N: Neomycin
NaCl: Sodium chloride
NGS: Next-generation sequencing
PBS: Phosphate-buffered saline
PCoA: Principal coordinates analysis
PEG: Polyethylene glycol
PPB: Potassium phosphate buffer
RSV: Respiratory syncytial virus
S. cerevisiae: Saccharomyces cerevisiae
S100a8: S100 calcium-binding protein A8
S100a9: S100 calcium-binding protein A9
SEM: Scanning electron microscopy
SEM: Standard error of the mean
SN: Bacterial supernatant
TEM: Transmission electron microscopy
Th17: T-helper 17
TNFα: Tumor necrosis factor α
Vanc, V: Vancomycin
YEPD: Yeast extract peptone dextrose
